# Motor unit number index (MUNIX) loss of 50% occurs in half the time of 50% functional loss according to the D50 disease progression model of ALS

**DOI:** 10.1038/s41598-023-30871-x

**Published:** 2023-03-09

**Authors:** Theresa Ebersbach, Annekathrin Roediger, Robert Steinbach, Martin Appelfeller, Anke Tuemmler, Beatrice Stubendorff, Hubertus Axer, Otto W. Witte, Julian Grosskreutz

**Affiliations:** 1 Department of Neurology, Jena University Hospital, Friedrich Schiller University, Am Klinikum 1, 07747 Jena, Germany; 2 Center for Healthy Ageing, Jena University Hospital, Jena, Germany; 3 Precision Neurology, University of Lübeck, Lübeck, Germany; 4 Cluster of Excellence Precision Medicine in Inflammation, University of Lübeck, Lübeck, Germany

**Keywords:** Motor neuron disease, Amyotrophic lateral sclerosis, Neurodegeneration, Biomarkers

## Abstract

Capturing disease progression in amyotrophic lateral sclerosis (ALS) is challenging and refinement of progression markers is urgently needed. This study introduces new motor unit number index (MUNIX), motor unit size index (MUSIX) and compound muscle action potential (CMAP) parameters called M50, MUSIX200 and CMAP50. M50 and CMAP50 indicate the time in months from symptom onset an ALS patient needs to lose 50% of MUNIX or CMAP in relation to the mean values of controls. MUSIX200 represents the time in months until doubling of the mean MUSIX of controls. We used MUNIX parameters of Musculi abductor pollicis brevis (APB), abductor digiti minimi (ADM) and tibialis anterior (TA) of 222 ALS patients. Embedded in the D50 disease progression model, disease aggressiveness and accumulation were analyzed separately. M50, CMAP50 and MUSIX200 significantly differed among disease aggressiveness subgroups (p < 0.001) regardless of disease accumulation. ALS patients with a low M50 had a significantly shorter survival compared to high M50 (median 32 versus 74 months). M50 preceded the loss of global function (median of about 14 months). M50, CMAP50 and MUSIX200 characterize the disease course in ALS in a new way and may be applied as early measures of disease progression.

## Introduction

Symptom onset, rate of disease progression and involvement of upper and lower motor neurons are highly variable in amyotrophic lateral sclerosis (ALS)^1,2^. It is crucial to develop innovative models allowing to objectively measure disease progression.

Motor unit number index (MUNIX) is a reliable tool to reflect the loss of lower motor neurons (LMNs) in ALS^3–5^. Based on surface electrodes, recording of a supramaximal compound muscle action potential (CMAP) and isometric contractions by the patient, an index reflecting functional LMNs is determined. The MUNIX technique is non-invasive and requires only a few minutes per muscle^6^. By the quotient of CMAP and MUNIX, motor unit size index (MUSIX) can be calculated, which is considered as a marker for reinnervation^7^. The technique was developed nearly two decades ago by Nandedkar and colleagues^8^. Since that time, MUNIX was examined and further developed in several studies in patients with motor neuron diseases^3,4,6,9–11^. Studies were performed with MUNIX sum scores of different muscles to represent the patients’ condition more holistically, mainly tibialis anterior (TA), abductor pollicis brevis (APB) and abductor digiti minimi (ADM) muscles were used. Decline in sum scores indicated LMN loss and correlated with the revised ALS functional rating scale (ALSFRS-R)^3,12,13^. However, given that MUNIX has proven to show decrease in functional LMN already in pre-symptomatic muscles in ALS it poses a promising biomarker^14^.

The heterogeneity of ALS is not limited to different phenotypes and symptom onset, but also affects disease duration and thus the timing of MUNIX measurements within the individual disease trajectory, much like any event in ALS e.g. time of diagnosis^2^. Briefly, the D50 model of disease progression can be used to address this heterogeneity because the model allows individual disease aggressiveness and accumulation to be considered separately in ALS patients^15–19^.

We hypothesize that using the new D50 disease progression model we can quantify the amount of time by which MUNIX, MUSIX and CMAP loss precede functional loss. To achieve this quantification, we developed a new approach derived from the ALSFRS-R based D50 disease progression model. Here, we describe the time in months to pre-defined changes in MUNIX parameters resulting in the new exploratory parameters M50, MUSIX200 and CMAP50.

## Materials

### Participants and methods

During clinical routine, 281 ALS patients received a MUNIX measurement in their individual disease course. A positive vote of the Jena University Hospital Ethics Committee (Nr. 3633-11/12) and informed consent of all participants were given in advance. The study was performed in accordance with ICH E6 (R2) guideline for good clinical practice. All MUNIX measurements of APB, ADM and TA were conducted at the Neurology Department of Jena University Hospital between 2013 and 2020 by trained and certified clinical neurophysiologists according to internationally approved MUNIX protocol and guideline^10,11^. In this case, we used the APB, ADM and TA, on the clinically less affected side to preserve read-out as long as possible (MUNIX guideline for recording signals and their analysis by Nandedkar et al.^10^). Previous studies suggest that these are the most reliable muscles because they had a lower interrater variability and lower coefficient of variation compared to other muscles^6,7,11^. The original MUNIX method and its computation were described in detail previously^8,10^. All MUNIX measurements were performed with the program Synergy on Nicolet EDX of Natus Neurology EMG Systems, which already contains a noise filter. The bandpass filter for CMAP was set to 3 Hz to 10 kHz and for surface interference patterns (SIP) to 10 Hz to 500 Hz. We used self-adhesive recording and reference electrodes, 20 × 15 mm (Ambu Neuroline 700). Investigators took care to record a maximal CMAP amplitude by moving the active electrode if necessary. For this purpose, the optimal electrode position was selected. Subsequently, the neurophysiologist instructed the patient to perform isometric contractions of different force levels (about 10%, 25%, 50%, 75% and maximal force of 100%) to record around 30 SIPs for 500 ms and took care to avoid additional muscle movements.

ALS patients were excluded if they did not fulfil the Gold Coast Criteria of ALS^20^ (n = 36, retrospectively), had MUNIX measurements on the clinically more affected side (n = 4), less than two ALSFRS-R questionnaires^21^ (n = 2) or juvenile ALS (n = 2). Few patients (n = 11) had a higher mean MUNIX than healthy controls, hence we could not calculate a M50 value because there was no decrease. Furthermore, all ALS patients with a M50 value significantly higher than normal life expectancy were excluded (n = 4). In total, 222 ALS patients with MUNIX were included in the study (see Table 1). The initial MUNIX measurement in the disease course of each patient was analyzed.

**Table 1:** Clinical and D50 model parameters of the ALS cohort.

	High	Intermediate	Low
	(0 ≤ D50 < 20)	(20 ≤ D50 < 40)	(D50 ≥ 40)
	n = 64	n = 92	n = 66
M50 in months	8.10 (5.81–11.4)	14.6 (10.8–20.7)	29.3 (19.8–43.2)
MUNIX APB	38.9 (2.00–91.8)	55.7 (14.9–100.3)	50.5 (15.0–100.5)
MUNIX TA	76.0 (37.1–108.6)	47.3 (2.00–92.3)	45.3 (2.00–99.1)
MUNIX ADM	73.6 (31.7–111.0)	71.3 (23.3–120.4)	66.3 (10.0–108.3)
MUSIX200 in months	9.04 (6.91–15.9)	16.8 (11.7–24.2)	33.1 (21.4–45.7)
MUSIX APB	97.8 (67.3–250.0)	89.3 (64.9–180.0)	88.2 (68.1–174.2)
MUSIX TA	49.3 (43.3–65.8)	59.4 (47.4–250.0)	73.2 (50.1–250.0)
MUSIX ADM	93.7 (72.4–133.7)	91.7 (72.0–130.9)	103.7 (75.4–194.3)
CMAP50 in months*	8.62 (6.37–14.2)	17.8 (12.0–24.4)	34.8 (21.3–58.6)
CMAP APB	3.78 (0.50–6.71)	4.66 (1.44–6.63)	4.49 (2.06–6.96)
CMAP TA	3.72 (1.96–5.22)	2.74 (0.50–4.95)	3.33 (0.50–5.38)
CMAP ADM	6.16 (3.10–8.45)	6.81 (3.09–9.12)	6.38 (1.11–9.59)
n of set small values	18/9/10	17/24/11	11/20/13
D50 disease progression model parameters			
rD50 at MUNIX	0.36 (0.23–0.45)	0.28 (0.18–0.41)	0.22 (0.12–0.34)
rD50 phase (I/II/III-IV)	21/33/10	35/46/11	36/28/2
D50 in months	13.8 (8.57–17.1)	28.0 (23.3–32.4)	61.5 (47.0–94.1)
Demographic and clinical parameters			
Age at MUNIX measurement	69.3 (58.9–75.5)	65.7 (58.9–69.9)	64.6 (57.6–70.6)
Gender (female/male)	30/34	39/53	28/38
Disease progression rate**	1.41 (0.91–2.39)	0.64 (0.47–0.80)	0.25 (0.16–0.38)
ALSFRS-R at MUNIX measurement**	36 (30–41)	38 (32–43)	40 (35–45)
n of ALSFRS-R observations	3 (3–5)	7 (4–10)	11 (6–17)
Disease duration at MUNIX	8.30 (6.09–12.4)	15.7 (10.4–24.3)	30.9 (19.6–45.1)
ALS phenotype			
Classic	33	53	42
Bulbar	30	34	15
Flail Arm	0	1	3
Flail Leg	0	0	2
Pyramidal	0	1	3
PLMN	1	3	1
Riluzole intake yes/no	56/8	84/8	58/8
Revised El Escorial criteria			
Definite	26	40	18
Probable	17	24	17
LSPR	21	26	25
Possible	0	2	6

Values are given as median and interquartile range or numbers.

*ALS* amyotrophic lateral sclerosis, *CMAP* compound muscle action potential, *MUNIX* motor unit number index, *MUSIX* motor unit size index, *LSPR* laboratory-supported probable, *PLMN* pure lower motor neuron. *Related to n = 62 in the high, n = 91 in the intermediate and n = 63 in the low aggressiveness subgroup, because 6 patients had no loss of function in comparison to the mean of CMAP of healthy controls. **Related to 199 of 222 ALS patients in whom the range between MUNIX and ALSFRS-R was 0 ± 4 weeks. Phenotypes in accordance to Chio et al.^25^.

The cohort of those who volunteered to be controls (n = 45) was the same as in our recent published article on MUNIX^19^. They were all older than 40 years and had no clinical evidence of peripheral or central nervous system diseases that could affect the measurements. Their MUNIX values were used to calculate a base level defined as 100 percent.

A sufficiently high value for CMAP (CMAP > 0.5 mV, MUNIX guidelines^10^) and MUNIX could not be obtained in every ALS patient. In these cases, we decided to set a MUNIX value of 2, a CMAP value of 0.5 mV and a MUSIX value of 250 (CMAP in µV/MUNIX = 500/2 = 250). With this approach, we avoided losing patients with advanced disease which would have created falsely high values in the remaining cohort.

### Application of the D50 disease progression model

MUNIX parameters were analyzed using the framework of the D50 disease progression model which was described in detail previously^15–19^. In essence, the model fits an individual sigmoidal decay curve of ALS patients with all available ALSFRS-R scores in disease course to create a mathematical abstraction of ALSFRS-R progression. All available ALSFRS-R scores of the patients were involved until October 2020 which were assessed in median every 2.8 months (interquartile range (IQR) 1.5–3.5 months).

Overall disease aggressiveness is then expressed by the parameter D50, which indicates the turning point of the sigmoid and equals the time in months patients take to lose 50% of their full clinical motor function (ALSFRS-R equals 24). The patient cohort can be divided into three subgroups of high (0 ≤ D50 < 20 months), intermediate (20 ≤ D50 < 40) and low (D50 ≥ 40) aggressiveness (see Table 1) as previously described^18,19^.

The second model parameter is relative D50 (rD50), which reflects disease accumulation normalized to D50. rD50 is thus a unitless scale where zero marks the onset of disease and 0.5 the point of 50% functional loss independent of the individual disease aggressiveness. This allows quantitative comparison between patients with vastly different forms of disease aggressiveness. rD50 can be stratified into different phases: the early (semi-)stable Phase I (0 ≤ rD50 < 0.25), the early progressive Phase II (0.25 ≤ rD50 < 0.5), and the late progressive and stable Phase III/IV (rD50 ≥ 0.5). By using these two parameters, disease aggressiveness (D50) and disease accumulation (rD50) can be quantitatively analyzed independently.

### M50, MUSIX200, CMAP50 and their application in the D50 disease progression model

Previous studies have shown that motor unit loss in ALS significantly precedes functional loss^3,14^. In order to estimate the time point of losing half of all available motor units in relationship to D50, we constructed the new parameter M50. M50 was calculated as the time point in months since symptom onset, ALS patients’ sum of the MUNIX values of the three muscles reached 50% of the average of the equivalent MUNIX sum of normal controls. For illustration, a sample calculation of M50 is presented in Supplementary Table 1. As previous studies showed an approximately linear decrease of MUNIX during this phase, we decided to use a linear estimation^3,12,14^.

In the same manner, the parameters CMAP50 (time in months until loss of 50% of the mean CMAP of controls) and MUSIX200 (time in months until doubling of the mean MUSIX of controls) were calculated.

### Statistical analysis

As our data did not show a normal distribution proven by Shapiro–Wilk test, we used the Kruskal–Wallis test with post-hoc pairwise Bonferroni correction for comparisons between three groups. Associations of variables were tested using the Spearman correlation coefficient r. Nominal variables were examined applying Chi-Square or Fisher–Freeman–Halton exact tests. The Kaplan–Meier method was applied for survival analyses. Subgroup comparisons were performed with the log-rank test. Statistical significance was defined as a p-value less than 0.05. Statistical analysis was performed with SPSS Statistics v27.0 (IBM, Chicago, IL, USA). GraphPad Prism v9.0 was used for illustrations (GraphPad Software, San Diego, CA, USA).

## Results

### Participant characteristics

The cohort of healthy controls had a mean age of 57.1 (standard deviation (SD) = 9.1) consisting of 32 females and 13 males. Mean MUNIX values and SD were as follows: APB 168.6 ± 58.6, ADM 154.4 ± 40.2 and TA 137.2 ± 28.9. These values of healthy controls are consistent with those from previous studies^22–24^.

Detailed clinical parameters and D50 disease progression parameters of the ALS cohort are described in Table 1. The median D50 was 28.4 months (IQR 18.4–45.5 months) and the median rD50 was 0.28 (IQR 0.18–0.41).

Age, gender, intake of riluzole and revised El Escorial categories were homogenously distributed across the D50 subgroups. In proportion, in the high aggressiveness disease group, there were significantly more patients with a bulbar phenotype compared to the intermediate and low disease aggressiveness group (p = 0.029).

Furthermore, patients with a high disease aggressiveness showed higher disease accumulation i.e. a more advanced disease phase (higher rD50) than patients in the intermediate and low disease aggressiveness group. This sampling shift is typical for cross-sectional cohorts, as previously described in studies using the D50 disease progression model^18^.

### M50, MUSIX200 and CMAP50 in the context of disease aggressiveness and accumulation

M50, MUSIX200 and CMAP50 were significantly associated with the D50 value representing individual disease aggressiveness (p < 0.001, r = 0.617/r = 0.601/r = 0.657). The Supplementary Fig. 1A–C show linear regression analyses of D50 with M50, MUSIX200 and CMAP50 respectively.

**Figure 1:**
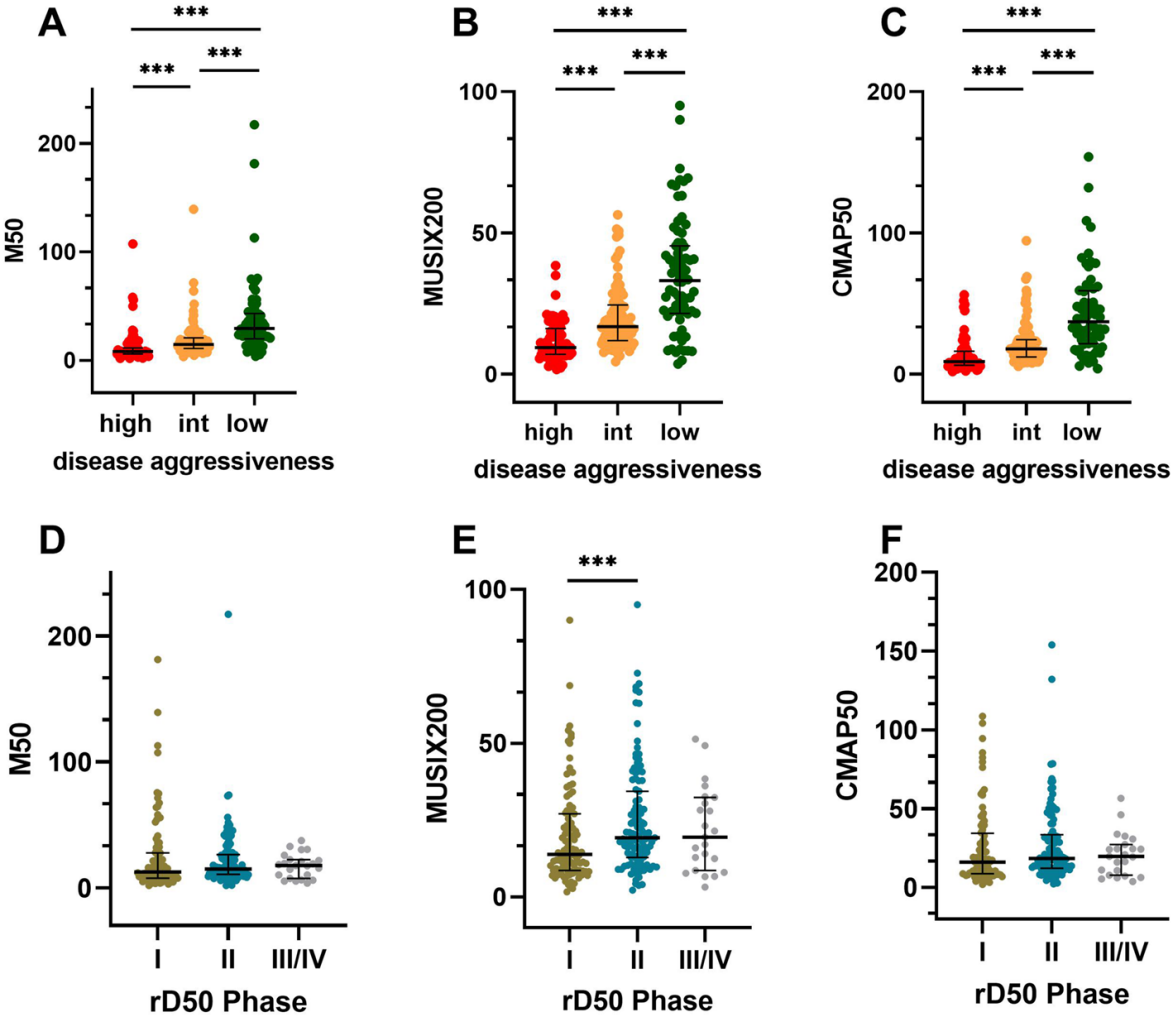
Scatterplots of M50, MUSIX200 and CMAP50. The ALS cohort was divided into three subgroups based on (**A**–**C**) disease aggressiveness and (**D**–**F**) disease accumulation expressed by rD50 phases. Bars indicate median and interquartile range. int, intermediate; ***p ≤ 0.001.

Dividing the ALS cohort into three subgroups of low (D50 ≥ 40 months), intermediate (20 ≤ D50 < 40) and high (0 ≤ D50 < 20) disease aggressiveness, M50, MUSIX200 and CMAP50 showed significant differences between all subgroups (p < 0.001; Fig. 1A–C).

In contrast to that, M50 and CMAP50 did not correlate with the rD50 phases and thus with disease accumulation, but MUSIX200 showed a significant association with rD50 (p = 0.01, r = 0.174). Correspondingly, there were no significant differences of M50, MUSIX200 or CMAP50 compared between subgroups of rD50 phases; except for MUSIX200 between Phase I and II (p < 0.001, Fig. 1D–F). M50 and D50 disease progression model parameters of rD50 subgroups are shown in Supplementary Table 2.

### M50 parameters and D50 show significant differences in survival

For analyses of survival with Kaplan–Meier and log-rank tests, we divided ALS participants based on D50, M50, MUSIX200 and CMAP50 medians respectively as a cut-off into low or high subgroups.

**Figure 2:**
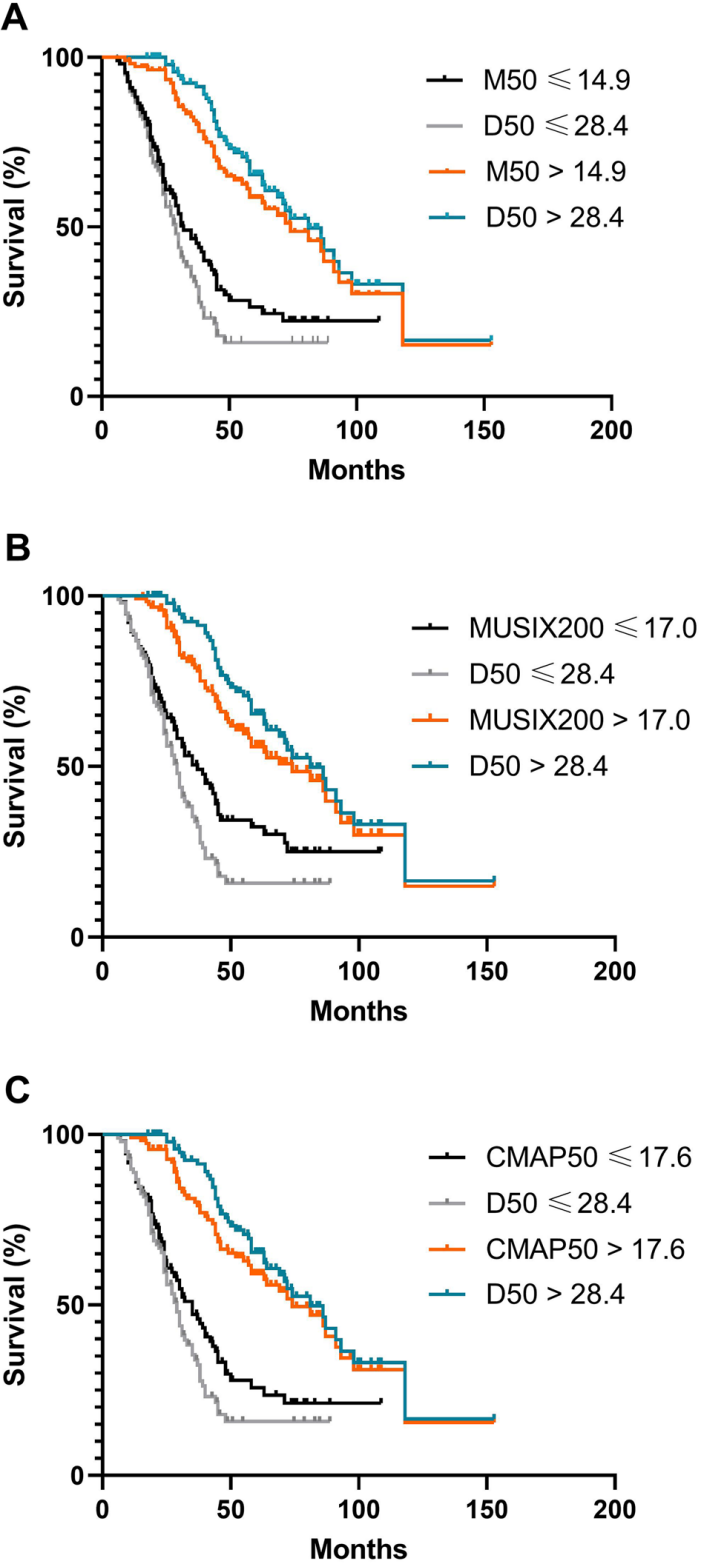
ALS patients were divided into two groups based on the medians (**A**) Comparison of M50 and D50 survival. Black line: M50 under 14.9 months, orange line M50 above 14.9 months, grey line: D50 under 28.42 months, middle blue line: D50 median above 28.42 months. Between low and high M50 and D50 group respectively, were significant differences (p < 0.001). (**B**) Comparison of MUSIX200 and D50 survival. Black line: MUSIX200 under 17.0 months, orange line MUSIX200 above 17.0 months, grey line: D50 under 28.42 months, middle blue line: D50 median above 28.42 months. Between low and high MUSIX200 and D50 group respectively, were significant differences (p < 0.001). (**C**) Comparison of CMAP50 and D50 survival. Black line: CMAP50 under 17.6 months, orange line CMAP50 above 17.6 months, grey line: D50 under 28.42 months, middle blue line: D50 median above 28.42 months. Between low and high CMAP50 and D50 group respectively, were significant differences (p < 0.001).

Splitting the ALS cohort into two groups based on the D50 median of 28.42 months revealed significant differences in survival (p < 0.001). The median survival of the low D50 group was 29 months (CI 25–33), whereas the median survival of the high D50 group was 86 months (CI 69–103) (Fig. 2).

For classification by M50 median (14.9 months), the median survival in the low M50 group was 32 months (95%-confidence interval CI 25–39), whereas the median survival in the high M50 group was 74 months (CI 53–95) with a significant difference (p < 0.001; Fig. 2A).

The ALS cohort had a median of MUSIX200 of 17.0 months. The subgroup with low MUSIX200 values had a median survival of 37 months (CI 28–46), the subgroup with high MUSIX200 values had a median survival of 69 months (CI 48–90), which was a significant difference (p < 0.001; Fig. 2B).

The median CMAP50 was 17.6 months. The low CMAP50 subgroup had a median survival of 35 months (CI 28–42), the high CMAP50 subgroup of 81 months (CI 58–104) with a significant difference between both (p < 0.001; Fig. 2C).

### M50 in relation to individual D50 values

Our results showed that M50 (median 14.9 months, IQR 9.0–26.5) preceded D50 (median 28.4 months, IQR 18.4–45.5) about 14 months which means that LMN function loss to the half (M50) measured by MUNIX dramatically preceded the loss of clinical function (D50).

For 186 ALS patients (= 84%) the M50 value precedes the D50 value by a median of 13.8 months. In 36 subjects only the D50 value was reached before the M50 value with a median interval of 9.8 months.

Nearly 75% of our cohort had M50 and D50 values either below or above the median (“same separation” group). The other 25% had “differing separation” of D50 and M50, that is either a low M50 (below the median) and a high D50 (above the median) or vice versa (Table 2). Between “same separation” and “differing separation” group there were no significant differences with respect to M50 and D50 parameters as well as age, sex, disease duration, phenotypes, and the medians of ALSFRS-R subscores. Looking more closely at the “differing separation” subgroups, patients in the “low M50 and high D50” subgroup were significantly earlier in individual disease progression in terms of a low rD50 median, the proportion of a bulbar phenotype was lower and the ALSFRS-R subscores of bulbar, fine motor and respiratory function were significantly higher.

**Table 2:** M50 and D50 median separation.

	Same separation	Differing separation	Differing separation		Sig.
			Low M50 and high D50	High M50 and low D50	
	n = 164	n = 58	n = 29	n = 29	
M50 in months	14.9 (8.19–29.7)	14.6 (10.7–22.0)	10.8 (8.10–13.0)	21.7 (16.8–27.7)	***
MUSIX200 in months	19.0 (10.1–34.0)	13.5 (8.83–19.7)	10.8 (8.19–13.7)	18.6 (13.2–23.3)	***
CMAP50 in months^$^	17.6 (9.69–38.6)	17.5 (11.2–23.7)	12.8 (8.85–15.4)	22.9 (18.3–30.8)	***
D50 disease progression model parameters					
rD50 at MUNIX	0.30 (0.20–0.41)	0.26 (0.13–0.39)	0.16 (0.10–0.26)	0.39 (0.23–0.51)	***
D50 in months	28.1 (17.3–48.2)	28.6 (20.3–38.7)	38.5 (31.0–47.5)	20.7 (15.6–25.6)	***
Demographic and clinical parameters					
Age at MUNIX	66.3 (58.7–72.4)	64.1 (57.5–72.4)	59.3 (54.9–66.4)	67.1 (58.7–75.4)	**
Gender (female/male)	74/90	23/35	7/22	16/13	
Disease duration at MUNIX in months	15.7 (8.73–30.7)	13.4 (8.88–19.3)	13.6 (9.52–16.9)	13.1 (7.89–23.9)	*
ALS phenotype					
Classic	92	36	23	13	**
Bulbar	57	22	6	16	**
Flail arm	4	0	0	0	
Flail leg	2	0	0	0	
Pyramidal	4	0	0	0	
PLMN	5	0	0	0	
ALSFRS-R Subscores					
Bulbar	11 (8–12)	10 (7–12)	11 (11–12)	7 (6–10)	***
Fine motor	9 (6–11)	10 (7–12)	10 (9–12)	8 (5–11)	**
Gross motor	9 (5–11)	9 (6–11)	9 (7–12)	8 (5–11)	
Respiratory	12 (10–12)	12 (10–12)	12 (11–12)	10 (8–12)	***

Values are given as median and interquartile range or numbers.

*ALS* amyotrophic lateral sclerosis, *ALSFRS-R* revised amyotrophic lateral sclerosis functional rating scale, *CMAP* compound muscle action potential, *MUNIX* motor unit number index, *MUSIX* motor unit size index, *LSPR* laboratory-supported probable, *PLMN* pure lower motor neuron, *Sig* significant difference between the “differing separation” subgroups (*p ≤ 0.05, **p ≤ 0.01, ***p ≤ 0.001) ^$^related to n = 160 at “same separation” and n = 56 at “differing separation”/n = 27 at “high M50 and low D50” because 6 patients had no loss of function in comparison to the mean of CMAP of healthy controls. Phenotype in accordance to Chio et al.^25^.

## Discussion

In this study we introduced the new MUNIX parameters M50, MUSIX200 and CMAP50 in first approximation to quantify the amount of time by which they precede loss of global function. The new approach to evaluation of MUNIX measurements and the advantages of these parameters are the consideration of the individual disease duration of ALS patients and the relation to the values of healthy controls in one parameter.

In previous MUNIX studies, only the absolute values between ALS and controls were compared in separate groups^12,13,23,26^. Furthermore, MUNIX was previously also considered using sum scores, which showed the clinical impairment and LMN loss in ALS, but did not include the disease duration, which ALS patients had up to the first time of MUNIX measurement^3,12,13,27^.

Furthermore, we analyzed these M50 parameter results with the D50 disease progression model, which allows consideration of disease aggressiveness and accumulation separately. This model divides the term “disease progression”, which had always been considered as a composite parameter in previous studies^3,12–14,26^, into the two parameters in a sigmoidal approach addressing the high heterogeneity in ALS patients^15–17^.

M50 correlated with the disease aggressiveness and was lowest in the subgroup with high disease aggressiveness. This also supports further investigations that MUNIX show a decline even in pre-symptomatic muscles in ALS patients, especially in high aggressive disease course^14^. Our observations are supported by the fact that these significant differences were also evident between disease aggressiveness subgroups for CMAP50 and MUSIX200. Together with M50, all three parameters moved in the same time range for each subgroup, with M50 always having the smallest value in median, indicating that M50 could be an early marker for LMN loss. MUSIX200 also showed its smallest median in the high aggressiveness subgroup, which may be interpreted as reinnervation to a doubling level relative to the baseline level of controls being significantly faster than in the other subgroups. These results suggest that faster LMN loss (to the half level of controls) is thus associated with a faster rate of reinnervation (to the doubling level of controls).

In contrast, the M50 and CMAP50 median remained stable across the different rD50 phases. They appear to be independent of disease accumulation. Regardless of the phase of disease accumulation in the individual ALS disease course, the time for loss of half of the baseline MUNIX or CMAP value of the controls was the same. In this context, we see the properties of CMAP as a supportive parameter to discuss our results of MUNIX. Interestingly, MUSIX200 showed a significant difference between Phase I and II. This indicates that the time to doubling of the MUSIX mean of the controls is smaller in Phase I, that is, reinnervation processes were probably performed faster on median in this early Phase I than in Phase II. This could suggest that especially in Phase I, motor neurons perform compensatory reinnervation processes rapidly to compensate for the loss as long as possible.

As described previously, faster disease progression in terms of a steeper fall of the ALSFRS-R is associated with a shorter survival^2^. In this study, using the D50 model, we were able to show the association of higher disease aggressiveness (lower D50) and significantly shorter survival time. Importantly, dividing the ALS cohort based on its M50 median into a low and a high M50 group, survival rates differed significantly. The median survival rate of ALS patients in the high M50 group was more than twice as high as in the low M50 group, which implicates a worser outcome in patients with higher LMN loss and could be a promising prognostic marker. Furthermore, survival classified by M50 could also serve as a surrogate marker in future therapeutic trials. Moreover, the M50 median of the ALS cohort preceded the D50 median by about 14 months underscoring the value of M50 as an early progression marker. This means, that the loss of LMN function in context of time span and healthy controls declined to 50% faster than global function determined by the parameter D50 based on observed ALSFRS-R questionnaires. As an addition to M50 median, the medians of CMAP50 and MUSIX200 were also reached prior to D50, supporting the assumption that MUNIX parameters precede the drop of global function, such as determined by the ALSFRS-R questionnaire or D50 parameters.

Almost 75% of the ALS cohort had the same separation of M50 and D50 medians (both below or above the median). Reasons for individual “differing separation” based on the medians of M50 versus D50 could be a bulbar or respiratory limitation, which is reflected in the ALSFRS-R and thus in D50, but not directly in MUNIX, as well as an only slight limitation of muscle strength in the early stages of the disease since the initial measurements of MUNIX were considered. In the differing separation group, “low M50 high D50”, the median ALSFRS-R subscores of gross and fine motor function are the same or higher than in the other subgroups, whereas the M50 value is already significantly lower in an early phase of the disease (low rD50), which underlines its value as an early disease marker.

Importantly the ALSFRS-R questionnaire is a patient related outcome measure which may be dependent on the personal assessment of the patient and prone to interrater differences, while MUNIX measurements are objective and quantitative neurophysiological measurements, which is clearly an advantage of the method.

In this study, we based M50 on APB, ADM and TA only as these muscles were the most reliable in our experience and also in the opinion of other investigators in other MUNIX studies^6,7,11^. However, the calculations might be easily extended to other muscles if necessary. As APB, TA and ADM are the mostly investigated muscles in MUNIX, results may be more reliable than those of less investigated muscles for less experienced investigators^7,11^. However, there might be an additional bias due to the limited number of investigated muscles.

As mentioned above, some patients did not achieve a CMAP or MUNIX value in all muscles in accordance with the MUNIX guideline^10^ due to the partly advanced muscle weakness. To rule out a shift to falsely high medians, we considered these values as small, fixed values to include patients in advanced disease stage. We would like to emphasize, that the key messages of this study remain the same regardless of the inclusion or exclusion of these minimum values.

The parameters M50, MUSIX200 and CMAP50 are not free of limitations. These parameters are based on the values of healthy controls from one single center. MUNIX, MUSIX and CMAP already had a large standard deviation in healthy controls, but several studies have shown that on average the values are within the same range^22–24^, so that the values can still provide a stable basis*.*

At time point of symptom onset, we have to consider that an unknown number of motor units are already gone in ALS as MUNIX decrease can be detected in pre-symptomatic muscles in ALS^14^. Which type of progression the decrease follows from pre-symptomatic stages is unclear; for the observation period in the individual disease, we were able to track, previous studies with MUNIX follow-up suggested a linear decrease^3,12,14^. Therefore, we decided to use a linear estimation. Future investigations with continuous MUNIX follow-up during symptomatic ALS disease course and in pre-symptomatic gene-carriers might be helpful to get a better understanding of the kinetics of motor units decrease in ALS muscles.

Calculation of M50 parameters from an average of healthy controls requires a significant drop of MUNIX values. The proportion of patients for whom no calculation was possible was less than 5% in this study. These were patients with a very slowly progressive disease or with measurements in very early disease stages after onset with bulbar phenotype.

To validate the results of our study, we need further multicenter investigations including a multicenter, age- and gender-matched healthy control cohort to establish normal range values for MUNIX in the mostly investigated muscles.

In conclusion, M50, MUSIX200 and CMAP50 assess the individual LMN function in ALS patients more detailed, as they include the disease duration and the relation to MUNIX, MUSIX and CMAP of healthy controls in one parameter, mitigating the disease heterogeneity, especially in the context of the D50 disease progression model. M50 parameters represent biomarkers for disease progression, which drop earlier on average than ALSFRS-R based markers. Therefore, M50, MUSIX200 and CMAP50 are promising parameters and should be validated and included in future MUNIX studies.

## Supplementary Information

Supplementary Information.

## Data Availability

The datasets generated and analyzed during the current study are available from the corresponding author on reasonable request.

## Abbreviations

ADM: Abductor digiti minimi muscle
ALS: Amyotrophic lateral sclerosis
ALSFRS-R: Revised amyotrophic lateral sclerosis functional rating scale
APB: Abductor pollicis brevis muscle
CI: 95%-Confidence interval
CMAP: Compound muscle action potential
EMG: Electromyography
IQR: Interquartile range
LMN: Lower motor neuron
LSPR: Laboratory-supported probable
MU: Motor unit
MUNIX: Motor unit number index
MUSIX: Motor unit size index
PLMN: Pure lower motor neuron
rD50: Relative D50
SD: Standard deviation
SIP: Surface interference pattern
TA: Tibialis anterior muscle
UMN: Upper motor neuron
