# Supplementary material for: Motor unit number index (MUNIX) loss of 50% occurs in half the time of 50% functional loss according to the D50 disease progression model of ALS
Source: Sci Rep. 2023 Mar 9;13:3981. doi: 10.1038/s41598-023-30871-x (PMC9998642; doi:10.1038/s41598-023-30871-x)
Supplement: Supplementary file 1 — Supplementary Information. [file 41598_2023_30871_MOESM1_ESM.pdf]

Motor unit number index (MUNIX) loss of 50% occurs in half the time of 50% functional loss according to the D50 disease progression model of ALS

Theresa Ebersbach[1,4], Annekathrin Roediger[1,4,*], Robert Steinbach[1], Martin Appelfeller[1], Anke Tuemmler[1], Beatrice Stubendorff [1], Hubertus Axer[1], Otto W. Witte[1,2] and Julian Grosskreutz[2,3]

1 Department of Neurology, Jena University Hospital, Friedrich Schiller University, Jena, Germany
2 Center for Healthy Ageing, Jena University Hospital, Jena, Germany
3 Precision Neurology, University of Lübeck, Lübeck, Germany
4 These authors contribute equally and share first authorship: Theresa Ebersbach and Annekathrin Roediger

*Correspondence to
Dr. med. Annekathrin Roediger
Department of Neurology, University Hospital Jena
Am Klinikum 1, 07747 Jena
E-Mail: annekathrin.roediger@med.uni-jena.de

Supplementary table 1 Sample calculation of M50 for one patient

**Mean values of healthy controls**		MUNIX APB	MUNIX TA	MUNIX ADM	
		168.58	137.22	154.43	
**MUNIX measurements of patient X**					
Symptom onset	MUNIX date	MUNIX APB	MUNIX TA	MUNIX ADM	
June 2016	June 12, 2018	37.89	78.79	80.20	
**Calculation for patient X**					Sum
In relation to the mean value of controls in %		22.48	57.42	51.93	131.83
Function from the mean of controls in % (131.83 divided by three measured values)					43.94
Loss from the mean of controls in %					**56.06**
Months from symptom onset to measurement					**24.38**
**M50** (months until loss of 50%): disease duration at MUNIX in months divided by loss from the mean of controls in % multiplied with 50		15.72	28.63	25.36	**21.75**

ADM, abductor digiti minimi; APB, abductor pollicis brevis; MUNIX, motor unit number index; TA, tibialis anterior.

Supplementary Table 2 Characteristics of the ALS cohort stratified by disease accumulation

		Disease accumulation		
	rD50 Phase	**I (0 ≤ rD50 < 0.25)**	**II (0.25 ≤ rD50 < 0.5)**	**III/IV (rD50 ≥ 0.5)**
		**n = 92**	**n = 107**	**n = 23**
M50 in months		12.7 (7.67 - 27.9)	15.0 (10.7 - 26.4)	17.8 (7.48 - 22.4)
MUNIX APB		73.3 (28.0 - 119.3)	38.9 (8.72 - 74.6)	2.00 (2.00 - 32.6)
MUNIX TA		86.9 (21.8 - 121.5)	44.2 (11.1 - 86.2)	36.7 (2.00 - 75.3)
MUNIX ADM		89.2 (44.5 - 124.6)	67.5 (22.1 - 109.8)	19.7 (8.17 - 53.1)
MUSIX200 in months		12.6 (8.32 - 22.6)	19.6 (12.8 - 34.9)	19.5 (8.70 - 32.4)
MUSIX APB		83.3 (59.8 - 139.4)	95.3 (71.7 - 207.2)	250.0 (88.5 - 250.0)
MUSIX TA		54.8 (45.5 - 105.2)	57.1 (47.5 - 200.1)	63.5 (45.9 - 250.0)
MUSIX ADM		88.8 (69.7 - 135.7)	93.8 (72.9 - 163.6)	123.1 (92.3 - 192.2)
CMAP50 in months*		15.6 (8.63 - 33.4)	18.3 (12.0 - 33.7)	19.7 (7.85 - 27.2)
CMAP APB		5.93 (3.47 - 7.98)	3.68 (1.51 - 6.36)	0.50 (0.50 - 3.57)
CMAP TA		4.47 (1.41 - 5.94)	2.84 (0.61 - 4.45)	2.32 (0.50 - 3.35)
CMAP ADM		7.34 (5.19 - 9.63)	6.15 (2.96 - 8.88)	2.54 (1.39 - 6.53)
D50		32.9 (20.7 - 61.6)	26.9 (17.6 - 41.7)	23.1 (8.56 - 29.4)
ALS phenotype	Classic	51	66	11
	Bulbar	33	35	11
	Flail Arm	3	1	0
	Flail Leg	2	0	0
	Pyramidal	3	1	0
	PLMN	0	4	1

Values are given as median and interquartile range or numbers, ALS, amyotrophic lateral sclerosis. CMAP, compound muscle action potential; MUNIX, motor unit number index; MUSIX motor unit size index; LSPR, laboratory-supported probable. PLMN, pure lower motor neuron. *related to n = 86 at Phase I because 6 patients had no loss of function in comparison to the mean of CMAP of healthy controls. Phenotype in accordance to Chio et al (Chio et al 2011).

Supplementary Figure 1

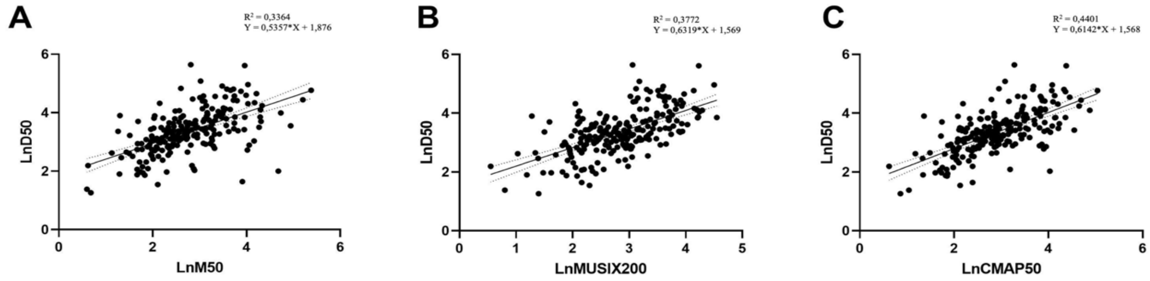

Linear regression of logarithmized (A) M50 and D50, (B) MUSIX200 and D50, (C) CMAP50 and D50.
Lines indicate regression and 95% confidence interval.
